# Impact of Embedded Memory Team in Primary Care

**DOI:** 10.1002/alz70858_103774

**Published:** 2025-12-26

**Authors:** K. Jennifer Ingram, David Carr, Genevieve Arsenault‐Lapierre, Safea Altef

**Affiliations:** ^1^ Trent University / Kawartha Centre, PETERBOROUGH, ON, Canada; ^2^ Medical Centre, Peterborough, ON, Canada; ^3^ McGill University, Montreal, QC, Canada

## Abstract

**Background:**

The Primary Care ‐ Embedded Memory Service (PC‐EMS) implements a dementia diagnostic framework, leverages existing community and Family Health Team (FHT) personnel to create a Memory Assessment Team in Primary Care (PC) sites. This enables all PC sites to have the best practice tools necessary to diagnose dementia with consistency and all patients to be managed by their own family physician. These changes are necessitated by increasing patient volumes with memory concerns, delays in diagnosis, declining numbers of PC physicians, and long wait times for specialists. The aim is to describe the pilot implementation and evaluate the outcomes of the PC‐EMS.

**Methodology:**

The PC‐EMS model trains existing nursing staff and Alzheimer Society assessors to form a specialized dementia intake assessment team. A PC specific Dementia Care Pathway, incorporated into the Electronic Medical Record (EMR), provides a coordinated diagnostic process. The evaluation consisted of (1) physicians’ knowledge, attitudes and practice (KAP) toward dementia care (2) patient and care partners satisfaction through surveys distributed once during the implementation, and (3) dementia quality as measured by retrospective review of PC‐EMS charts compared to pre‐intervention control group charts.

**Results:**

12 physicians and Nurse Practitioners of 15 referring physicians, 32 patients, and 27 care partners completed the surveys. 49 patients’ charts and 50 control charts were reviewed. Patient and care partners’ satisfaction and physicians’ KAP scores were high. More importantly, documentation of key quality indicators (IADLs, BADLs, caregiver experience, and cognitive testing), time to follow‐up by the patient's PC Physician, and use of specialist resources improved. The model addresses increasing patient volumes, ensures care partners’ involvement, and improves the quality and consistency of charting.

**Conclusion:**

The pilot evaluation of the PC‐EMS at the Medical Centre demonstrates an innovative program for dementia diagnosis within PC settings, which can be easily replicated and expanded to address future requirements for prompt person‐centred dementia diagnosis. The results inform policy development and system change to ensure care partners and persons with dementia are jointly involved with their familiar PC physician in the process of establishing a diagnosis of dementia and initiating care.

